# COVID-19 Rapid Antigen Test as Screening Strategy at Points of Entry: Experience in Lazio Region, Central Italy, August–October 2020

**DOI:** 10.3390/biom11030425

**Published:** 2021-03-13

**Authors:** Francesca Colavita, Francesco Vairo, Silvia Meschi, Maria Beatrice Valli, Eleonora Lalle, Concetta Castilletti, Danilo Fusco, Giuseppe Spiga, Pierluigi Bartoletti, Simona Ursino, Maurizio Sanguinetti, Antonino Di Caro, Francesco Vaia, Giuseppe Ippolito, Maria Rosaria Capobianchi

**Affiliations:** 1 National Institute for Infectious Diseases, “Lazzaro Spallanzani” IRCCS, 00149 Rome, Italy; francesca.colavita@inmi.it (F.C.); francesco.vairo@inmi.it (F.V.); silvia.meschi@inmi.it (S.M.); mariabeatrice.valli@inmi.it (M.B.V.); eleonora.lalle@inmi.it (E.L.); concetta.castilletti@inmi.it (C.C.); antonino.dicaro@inmi.it (A.D.C.); Francesco.vaia@inmi.it (F.V.); giuseppe.ippolito@inmi.it (G.I.); 2 Lazio Regional Health Service, 00145 Rome, Italy; dfusco@regione.lazio.it (D.F.); gspiga@regione.lazio.it (G.S.); 3 Regional Special Unit for Community Health Care (USCAR), National Institute for Infectious Diseases “Lazzaro Spallanzani” IRCCS, 00149 Rome, Italy; pl.bartoletti@gmail.com; 4 Local Health Authority-Roma 4, Civitavecchia, 00153 Rome, Italy; simona.ursino@aslroma4.it; 5 Fondazione Policlinico Universitario “A. Gemelli” IRCCS, 00168 Rome, Italy; maurizio.sanguinetti@unicatt.it

**Keywords:** SARS-CoV-2, rapid antigen test, point of entry, Italy

## Abstract

COVID-19 pandemic is a dramatic health, social and economic global challenge. There is urgent need to maximize testing capacity. Rapid Antigen Tests (RAT) represent good candidates for point-of-care and mass surveillance testing to rapidly identify SARS-CoV-2-infected people, counterbalancing lower sensitivity vs. gold standard molecular tests with fast results and possible recurrent testing. We describe the results obtained with the testing algorithm implemented at points of entry (airports and ports) in the Lazio Region (Italy), using the STANDARD F COVID-19 Antigen Fluorescence ImmunoAssay (FIA), followed by molecular confirmation of FIA-positive samples. From mid-August to mid-October 2020, 73,643 RAT were reported to the Regional Surveillance Information System for travelers at points of entry in Lazio Region. Of these, 1176 (1.6%) were FIA-positive, and the proportion of RT-PCR-confirmed samples was 40.5%. Our data show that the probability of confirmation was directly dependent from the semi-quantitative FIA results. In addition, the molecularly confirmed samples were those with high levels of virus and that were actually harboring infectious virus. These results support public health strategies based on early mass screening campaigns by RAT in settings where molecular testing is not feasible or easily accessible, such as points of entry. This approach would contribute to promptly controlling viral spread through travel, which is now of particular concern due to the spread of SARS-CoV-2 variants.

## 1. Introduction

As of 11th February 2021, the COVID-19 pandemic is continuing to spread worldwide, accounting for a total of over 106,991,090 cases and 2,347,015 deaths, with high impact on healthcare systems and devastating global socio-economic consequences [1]. With COVID-19 cases accelerating towards a second wave for many countries and a further overburden on health care systems and laboratories, there is an urgent need to expand testing capacity, in order to quickly identify as many SARS-CoV-2-positive persons as possible in order to control infection transmission [2,3,4].

The nucleic acid amplification test (NAAT) is the gold standard for the diagnosis of SARS-CoV-2 infection; however, it is laborious, expensive, and faces the challenge of a shortage of reagent supplies [4,5,6]. Rapid antigen tests (RAT) represent a good option for mass testing and for rapidly capturing individuals that are potentially more infectious, especially in decentralized settings, or in those scenarios where molecular testing is not feasible or easily accessible. In fact, despite their lower sensitivity, they are able to identify current infections during the most contagious phase and are faster, simpler-to-use, and less expensive than NAAT [7,8]. Overall, these tests can ultimately help to relieve the pressure on healthcare systems and support public health strategies to control virus spread. The variability of the clinical performance is one of the limiting factors of RAT [7,9]; however, their use may be of a greater benefit when compared to the risks associated with no testing, especially for effective and sustainable surveillance regimens [3,10]. The global request for ‘test, test, test’ has increased the attention and expectations directed toward these tools. Debate is ongoing among international public health agencies and national authorities to define the most reliable way to exploit RAT [5,11,12]. As a matter of fact, the manner in which to incorporate RAT in diagnostic algorithms and in public health strategies is the primary aspect that needs to be addressed in order to balance the benefits and limitations of these tests.

Here, we describe the preliminary data from our experience in implementing RAT at points of entry (PoE) in Rome, Italy, between 17th of August to 15th of October 2020.

## 2. Materials and Methods

In August 2020, following the end of the national lockdown, the re-opening of borders and the intensification of citizen travels due to summer holidays, Italian authorities strengthened border surveillance [13]. Therefore, on 17th of August, in the Lazio region, on-site screening by SARS-CoV-2 RAT was implemented at the international airports in Rome (“Leonardo da Vinci International Airport”, Fiumicino, and “Ciampino–G. B. Pastine International Airport”, Ciampino), and at the port of Civitavecchia (Rome) for those ships returning from the Sardinia region, for travelers returning from high-incidence foreign countries, as well as from the Sardinia region (Italy). Antigenic testing was voluntary, and the sample collection and RAT execution was performed by trained health care workers deployed at the PoE (Regional Special Unit for Community Health Care, USCAR). The RAT used was the STANDARD F COVID-19 Ag Fluorescence ImmunoAssay (FIA, SD Biosensor, Suwon, Korea). This test detects viral nucleoprotein (N) directly from nasopharyngeal swab (NPS); the interpretation of results is performed after 30 min incubation using an automatic fluorescence reader (STANDARD F100, SD Biosensor, Suwon, Korea) that gives a cut-off index (COI) as measure of fluorescence signal detected in relation to the presence of viral antigen; COI ≥ 1 is interpreted as positive for the SARS-CoV-2 N antigen. Based on the sensitivity and specificity of this assay, established on a preliminary validation study [14], assuming 1% prevalence, positive and negative predictive values (PPV and NPV) of this test were estimated to be 23.3% (95% CI: 10.1–45.0) and 99.5% (95% CI: 99.4–99.6), respectively. Based on this assumption, the adopted algorithm was to confirm only RAT-positive results. For NAAT confirmation, a second NPS was collected in UTM medium (COPAN, Murrieta, CA, USA.) immediately after the RAT results and readily sent to the Laboratory of Virology of the National Institute for Infectious Diseases “L. Spallanzani” (INMI). Different Real-Time RT-PCR (RT-PCR) platforms available for the routine COVID-19 diagnosis (DiaSorin Simplexa^®^ COVID-19 Direct, DiaSorin, Saluggia, Italy; Roche Diagnostics Cobas^®^ SARS-CoV-2, Roche, Basilea, Switzerland; Abbott RealTime SARS-CoV-2, Abbott, Des Plaines, IL, USA) were used as molecular confirmatory tests. In the case of negativity by RAT, appropriate communication regarding the mandatory caution in the interpretation of the results was adopted, including recommendation of continued use of transmission prevention measures, such as mask wearing and social distancing. Virus culture was performed in BSL-3 laboratory on selected RT-PCR confirmed samples, using Vero E6 cell line, as previously described [15]. Results of each RAT were recorded at the testing site through an electronic register and, subsequently, uploaded to the Regional Surveillance Information System established by the regional health authority. Anonymized data were extracted and analyzed using STATA 14 statistical software. Laboratory data (SARS-CoV-2 RT-PCR results) were recorded on the Laboratory Information System in use at the Laboratory of Virology of INMI; when available, COI values obtained from the RAT reader at the testing site were matched with the results of molecular tests used for laboratory confirmation. For comparison between COI and viral RNA level, only samples tested with molecular platforms addressing ORF1 as viral genome target were used, in order to allow homogeneity of RT-PCR results. SARS-CoV-2 RNA copies number used for correlation analysis between COI and viral RNA load, was inferred by extrapolation, using serial dilutions of a SARS-CoV-2 isolate (2019nCoV/Italy INMI1, GISAID accession number EPI_ISL_410546) quantified on the basis of a standard curve for SARS-CoV-2 E gene. Spearman correlation test was performed using GraphPad Prism version 8.00 (GraphPad Software, San Diego, CA, USA). ROC curve analysis and PPV/NPV were calculated using the MedCalc statistical software (MedCalc Software Ltd., Ostend, Belgium).

## 3. Results

From the 17th of August to 15th of October 2020, a total of 73,643 RAT, performed on travelers at the PoE, were reported to the Regional Surveillance Information System. Of these, 72,467 were FIA-negative, while 1176 (1.6%) were positive. On the Regional Surveillance Information System, matched NAAT confirmation results were available for 941 of the 1176 RAT positive samples, resulting in 381 (40.5%) RT-PCR positive confirmations. The proportion of RAT confirmed results by NAAT was well within the 95% CI of the expected proportion, on the basis of the PPV calculated for 1% prevalence [14].

**Figure 1:**
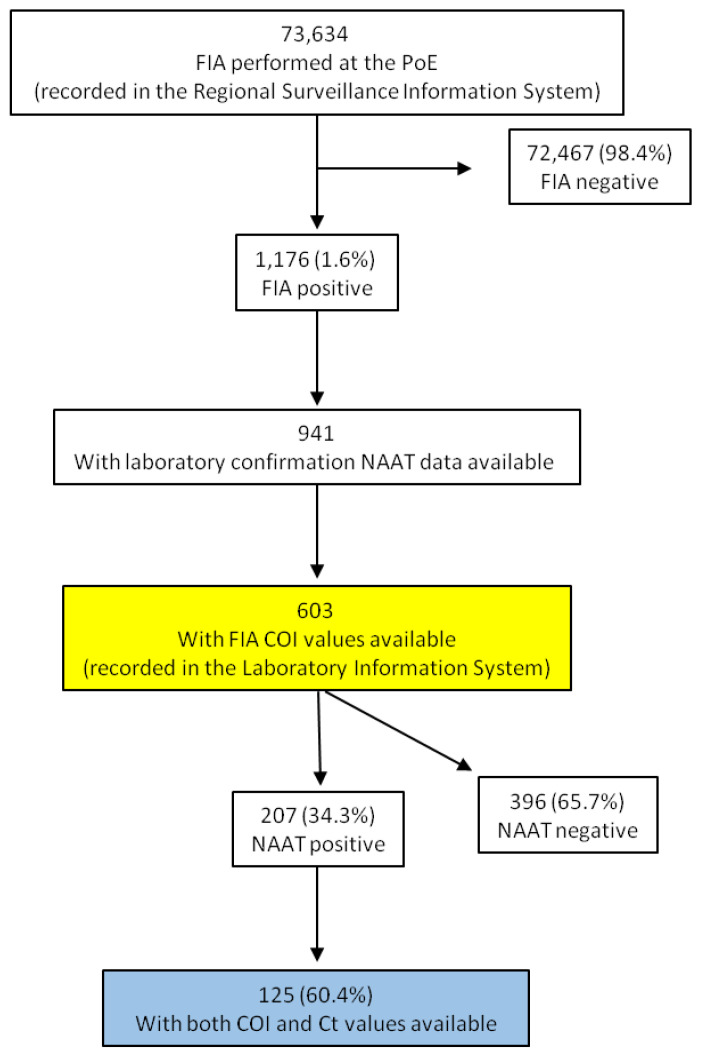
Flow chart of the analysis performed on the records from COVID-19 FIA testing at PoE and NAAT laboratory confirmation. The yellow and blue boxes correspond to the data analyzed in Table 1 and Figure 2, respectively.

The analysis of the semi-quantitative data from the tools employed in this study was performed on a sub-set (*n* = 603) of samples from subjects tested positive by RAT with available COI values (Figure 1).

**Figure 2:**
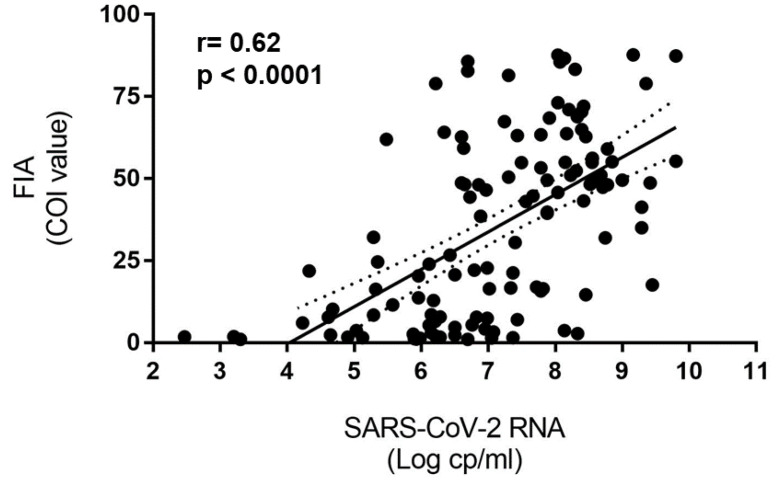
Correlation between SARS-CoV-2 RNA copies number and COI obtained on confirmed SARS-CoV-2 positive samples with available information for both parameters (*n* = 125, as in blue box of Figure 1).

The COI range of this sub-set was 1.01–87.7. Of these samples, 207 (34.3%) were confirmed to be positive by SARS-COV-2 RT-PCR. For 125 samples, both Ct ORF1 and COI values were available. The median Ct value was 19.8 (range 11.1–34), corresponding to high viral loads in NPS which are potentially more infectious [5]. Viral culture on VERO E6 cells was attempted on 10 of these NPS were positive by RAT and confirmed by SARS-COV-2 RT-PCR (Ct range: 11.4–19.1); replication-competent virus was recovered from all of these samples, supporting the potential for SARS-CoV-2 transmission. In addition, a significant correlation (*r* = −0.60; *p* < 0.0001) was observed between the Ct values, which are surrogate markers of viral load (lower Ct value corresponds to high viral load), and COI values resulted from the RAT, which represent the extent of the antigen detection. As a matter of fact, in Figure 2, the correlation between COI values and RNA copy number, inferred from a standard curve based on a virus preparation with known RNA copy content, is shown, perfectly overlapping with the correlation coefficient obtained with Ct values. No significant correlation was instead found when the analysis was restricted to samples with low (below 3) COI values (*r* = 0.1, *p* = 0.71).

In fact, the median COI value of the RAT not confirmed by SARS-CoV-2 RT-PCR (*n* = 397) was very low (1.4, range: 1.0–15.0). We then stratified the RT-PCR result confirmation rate according to the COI obtained by RAT. As shown in Table 1, the percentage of confirmed RAT results was strongly dependent on the COI value, ranging from 81.6% when the COI threshold was set at 3 to 100% when the COI threshold was set at 20. 

**Figure 3:**
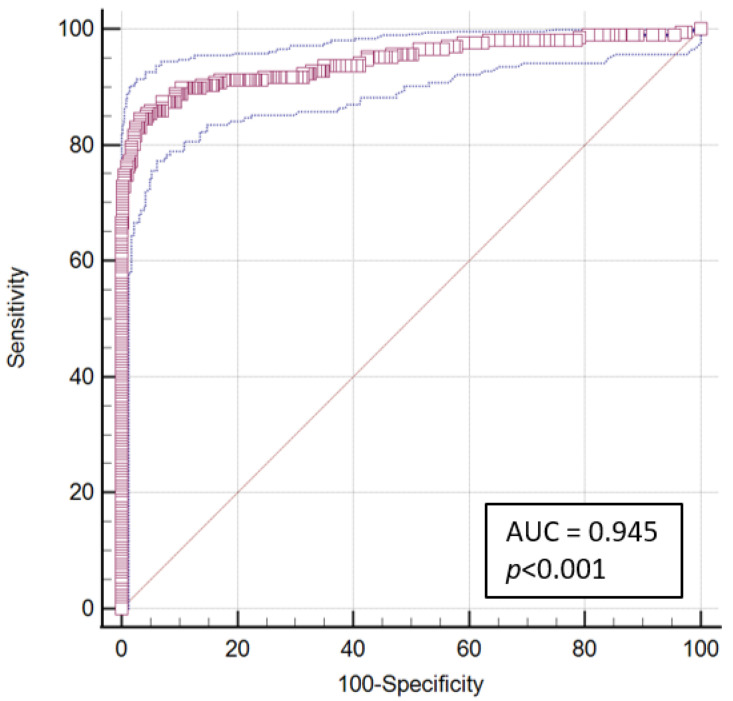
ROC curve analysis performed for FIA to detect COVID-19 NAAT confirmed cases (*n* = 603, as in yellow box of Figure 1).

More precisely, positive RAT with COI ≥ 10 were confirmed in 98.7% of cases with only 1.3% of RAT false positive results, while lower cut-off showed higher percentage of RAT false positivity. This is confirmed also by the ROC analysis performed on the data from the 603 subjects tested positive by RAT with available COI values (Figure 3). The area under the ROC curve (AUC) was 0.94 (95% CI: 0.9 to 1.0; *p* < 0.001); the optimal criterion obtained with a prevalence of 1% was COI > 15.02 (Sensitivity: 66.7, 95% CI 59.8 to 73.0; Specificity: 100.0, 95% CI 99.1 to 100.0). More conservative COI thresholds may be established to increase the chance of identifying true SARS-CoV-2 positive samples with low detrimental effect on specificity (e.g., 10, Sensitivity: 72.9, 95% CI 66.4 to 78.9; Specificity: 99.5, 95% CI 98.2 to 99.9).

## 4. Discussion

In this study, we evaluated the results of the SARS-CoV-2 testing strategy at PoE in Lazio Region using RAT in order to reduce the risk of importing cases and limit new chains of transmission. The test was voluntary, and the tool implemented was the STANDARD F COVID-19 Ag FIA (SD Biosensor, Suwon, Korea), characterized by 30 min until results and the use of an automatic user-friendly reader that guarantees objective interpretation of the results. More than 70,000 travelers arriving at airports and port in the Lazio region were screened by RAT between 17th of August and 15th of October 2020, and a small proportion (1.6%) tested positive for the SARS-CoV-2 antigen. The proportion of samples confirmed with RT-PCR was 40.5%, with almost 60% of those being false positives, as is to be expected in low-prevalence settings [5,12].

As for other RAT, a previous study showed that STANDARD F COVID-19 Ag FIA is highly specific (98.4%, 95% CI: 96.0% to 99.6%) for SARS–CoV-2 Ag detection in NPS, and highly sensitive (95.2%, 95% CI: 76.2% to 99.9%) for those samples with Ct values lower than 25, which are harbored by patients with high viral load, who are more likely to be expected to be able to transmit the infection [14,16,17]. In our experience, high viral load values (median Ct value = 19.9) were observed in samples confirmed to be positive by NAAT. More importantly, our study shows that clinical samples showing positive results by RAT and confirmed by NAAT harbor infectious virus. To our knowledge, this is the first report showing a linear correlation between viral loads in NPS (measured as Ct values) and extent of viral antigen detection (measured as COI values). As a consequence, the proportion of RAT samples confirmed by a molecular test was extremely high (98.7%) in those samples with COI ≥ 10, reaching 100% with COI ≥ 20. This information may be of assistance for the reliable diagnostic interpretation of this specific tool and may be useful for a potential reassessment of the diagnostic algorithms of RAT confirmation by NAAT. In fact, on the basis of the proportion of cases confirmed by NAAT according to their COI value and according to the overall ROC analysis, a threshold (e.g., COI ≥ 15, or ≥ 10 as a more conservative cut-off for NAAT confirmation, in order to maximize the identification of true COVID-19 positive cases) may be established to exempt molecular testing when the expected confirmation rate exceeds 95%. Following this principle, it is possible that specific thresholds may also be evaluated for other semi-quantitative RAT, prompting not only a consequent earlier implementation of public health measures, but also a potential advantage for the NAAT laboratory workload and demand in terms of staff effort and reagent supplies, ultimately helping to relieve the pressure on healthcare systems [3,4,11,16].

A limitation of the present study is that RAT with negative results at PoE did not undergo RT-PCR testing, so it was not possible to estimate the proportion of PCR-positive samples missed by RAT. However, in our opinion, though RAT are substantially less sensitive than NAAT [8], they are worth being integrated into COVID-19 outbreak management programs, as they may contribute to the prompt isolation of highly infectious cases who would otherwise be lost. This aspect highlights the role of PoE testing as a pillar of outbreak control, especially in the current time, when the spread of SARS-CoV-2 variants through travel is raising particular concern worldwide [11,18]. In fact, the use of RAT may be particularly advantageous to the early minimization of the risk of the virus spreading, especially when and where there is no immediate access to RT-PCR testing, or where this cannot be feasible, such as for mass and frequent testing or in certain field settings. In addition, RAT use could also represent a suitable screening tool for prompt cluster investigation and in specific cohorts such as asymptomatic contacts of COVID-19 confirmed cases, pauci-symptomatic patients with no epidemiological link, and travelers with no symptoms [11]. Of course, public-awareness campaigns must also communicate that a negative RAT does not necessarily implies a clean bill of health, and continued social distancing and mask wearing needs to be recommended. In addition, negative RAT results in symptomatic patients should not be considered definitive, and molecular tests are required for a more reliable diagnostic assessment. Finally, it is necessary to balance clinical diagnostic performances of the tests, testing sustainability (i.e., cost, staff demand, technology and infrastructures, time to results), public health implications, and socio-economic consequences, which can also derive from delay in diagnostic response [3,19].

## 5. Conclusions

Our data show that the probability of molecular confirmation of positive results by STANDARD F COVID-19 Ag FIA is directly dependent from the semi-quantitative data of this specific RAT, and that the molecularly confirmed samples actually harbor infectious virus. These results support public health strategies based on early mass screening campaigns, using rapid and simple point-of-care tools, in settings where molecular testing is not feasible or easily accessible, such as the points of entry. This approach would contribute to promptly controlling viral spread through travel, which is now of particular concern due to the emergence of SARS-CoV-2 variants.

## Figures and Tables

**Table 1:** Percentage of confirmed FIA-positive results by SARS-CoV-2 RT-PCR according to the COI values (*n* = 603, as in yellow box of Figure 1).

COI	RT-PCR Positive/N (% TP)	% FP by FIA
≥1	207/603 (34.3%)	65.7%
≥3	186/228 (81.6%)	18.4%
≥5	175/188 (93.1%)	6.9%
≥8	159/165 (96.4%)	3.6%
≥10	152/154 (98.7%)	1.3%
≥15	138/139 (99.3%)	0.7%
≥20	127/127 (100%)	0.0%

Abbreviation: COI, cut-off index; TP, true positive; FP, false positive.

## Data Availability

The data used and/or analyzed during the current study are available, only for sections non-infringing personal information, from the corresponding author on reasonable request.

## References

[1] World Health Organization WHO Coronavirus Disease (COVID-19) Dashboard. https://covid19.who.int/.

[2] Rubin R. (2020). The challenges of expanding rapid tests to curb COVID-19. JAMA.

[3] Mina M.J., Parker R., Larremore D.B. (2020). Rethinking Covid-19 test sensitivity—A strategy for containment. N. Engl. J. Med..

[4] Vandenberg O., Martiny D., Rochas O., van Belkum A., Kozlakidis Z. (2020). Considerations for diagnostic COVID-19 tests. Nat. Rev. Microbiol..

[5] WHO Antigen-Detection in the Diagnosis of SARS-CoV-2 Infection Using Rapid Immunoassays: Interim Guidance. https://apps.who.int/iris/handle/10665/334253.

[6] Fomsgaard A.S., Rosenstierne M.W. (2020). An alternative workflow for molecular detection of SARS-CoV-2—Escape from the NA extraction kit-shortage, Copenhagen, Denmark, March 2020. Eurosurveillance.

[7] Mak G.C., Cheng P.K., Lau S.S., Wong K.K., Lau C.S., Lam E.T., Chan R.C., Tsang D.N. (2020). Evaluation of rapid antigen test for detection of SARS-CoV-2 virus. J. Clin. Virol..

[8] Lai C.K.C., Lam W. (2021). Laboratory testing for the diagnosis of COVID-19. Biochem. Biophys. Res. Commun..

[9] Krüger L.J., Gaeddert M., Köppel L., Brümmer L.E., Gottschalk C., Miranda I.B., Schnitzler P., Kraeusslich H.G., Lindner A., Nikolai O. (2020). Evaluation of the accuracy, ease of use and limit of detection of novel, rapid, antigen-detecting point-of-care diagnostics for SARS-CoV-2. medRxiv.

[10] Nalumansi A., Lutalo T., Kayiwa J., Watera C., Balinandi S., Kiconco J., Nakaseegu J., Olara D., Odwilo E., Serwanga J. (2020). Field evaluation of the performance of a SARS-CoV-2 antigen rapid diagnostic test in Uganda using nasopharyngeal samples. Int. J. Infect. Dis..

[11] Istituto Superiore di Sanità (2020). Nota tecnica ad interim. Test di laboratorio per SARS-CoV-2 e loro uso in sanità pubblica.

[12] Center for Disease Control and Prevention (CDC) Interim Guidance for Rapid Antigen Testing for SARS-CoV-2. https://www.cdc.gov/coronavirus/2019-ncov/lab/resources/antigen-tests-guidelines.html.

[13] Ministero della Salute (2020). Disposizioni Attuative del Decreto-Legge 25 Marzo 2020, n. 19, Recante Misure Urgenti per Fronteggiare L’Emergenza Epidemiologica da COVID-19, e del Decreto-Legge 16 Maggio 2020, n. 33, Recante Ulteriori Misure Urgenti per Fronteggiare l’Emergenza Epidemiologica da COVID-19, 20A02717.

[14] Liotti F.M., Menchinelli G., Lalle E., Palucci I., Marchetti S., Colavita F. (2020). Performance of a novel diagnostic assay for rapid SARS-CoV-2 antigen detection in nasopharynx samples. Clin. Microbiol. Infect..

[15] Colavita F., Lapa D., Carletti F., Lalle E., Messina F., Rueca M. (2020). Virological characterization of the first two COVID-19 patients diagnosed in Italy: Phylogenetic analysis, virus shedding profile from different body sites and antibody response kinetics. Open Forum Infect. Dis..

[16] Cerutti F., Burdino E., Milia M.G., Allice T., Gregori G., Bruzzone B., Ghisetti V. (2020). Urgent need of rapid tests for SARS CoV-2 antigen detection: Evaluation of the SD-Biosensor antigen test for SARS-CoV-2. J. Clin. Virol..

[17] Porte L., Legarraga P., Vollrath V., Aguilera X., Munita J.M., Araos R. (2020). Evaluation of a novel antigen-based rapid detection test for the diagnosis of SARS-CoV-2 in respiratory samples. Int. J. Infect. Dis..

[18] World Health Organization (2020). COVID-19 Strategic Preparedness and Response Plan Operational Planning Guidelines to Support Country Preparedness and Response. https://www.who.int/docs/default-source/coronaviruse/covid-19-sprp-unct-guidelines.pdf?sfvrsn=81ff43d8_4.

[19] Pettengill M.A., McAdam A.J. (2020). Can we test our way out of the COVID-19 pandemic?. J. Clin. Microbiol..

